# Place of death in haematological malignancy: variations by disease sub-type and time from diagnosis to death

**DOI:** 10.1186/1472-684X-12-42

**Published:** 2013-11-19

**Authors:** Debra A Howell, Han-I Wang, Alexandra G Smith, Martin R Howard, Russell D Patmore, Eve Roman

**Affiliations:** 1 Department of Health Sciences, University of York, York YO10 5DD, UK; 2 York Teaching Hospital NHS Foundation Trust, Wigginton Road, York YO31 8HE, UK; 3 Queens Centre for Oncology, Castle Hill Hospital, Cottingham, Hull HU16 5JQ, UK

**Keywords:** Leukaemia, Lymphoma, Myeloma, End-of-life, Dying

## Abstract

**Background:**

The reasons patients with haematological malignancies die in hospital more often than those with other cancers is the subject of much speculation. We examined variations in place of death by disease sub-type and time from diagnosis to death, to identify groups of ‘at-risk’ patients.

**Methods:**

The study is based in the United Kingdom within the infrastructure of the Haematological Malignancy Research Network (HMRN), a large on-going population-based cohort including all patients newly diagnosed with haematological malignancies in the north of England. Diagnostic, demographic, prognostic, treatment and outcome data are collected for each patient and individuals are ‘flagged’ for death. This study includes all adults (≥18 years) diagnosed 1st September 2004 to 31st August 2010 (n = 10,325), focussing on those who died on/before 31st August 2012 (n = 4829).

**Results:**

Most deaths occurred in hospital (65.9%), followed by home (15.6%), nursing home (11%) and hospice (7.5%) and there was little variation by diagnostic sub-type overall. Differences in place of death were, however, observed by time from diagnosis to death, and this was closely related to sub-type; 87.7% of deaths within a month of diagnosis happened in hospital and these largely occurred in patients with acute myeloid leukaemia, diffuse large B-cell lymphoma and myeloma. Patients surviving longer, and particularly beyond 1 year, were less likely to die in hospital and this corresponded with an increase in the proportion of home deaths.

**Conclusions:**

Time from diagnosis to death was clearly a major determinant of place of death and many patients that died within three months of diagnosis did so in hospital. This was closely related to disease sub-type, with early deaths occurring most notable in the more aggressive diseases. This is likely to be due to a combination of factors including acute presentation, rapid disease progression without transition to a palliative approach to care and complications of treatment. Nonetheless, hospital deaths also occurred frequently in indolent diseases, suggesting that other factors were likely to contribute to the large proportion of hospital deaths overall. More evidence is needed to fully understand these complex cancers.

## Background

Many studies report that, given the choice, most people would prefer to die at home [1-3]. However, it is well recognised that patients with haematological malignancies are more likely to die in hospital than those with other cancers or non-malignant diseases [4-6]. This has particular implications for haematology patients, as it means they may not be dying in their preferred place. Within the United Kingdom (UK), the promotion of quality care for people approaching the end of their lives has been driven by the National Health Service (NHS) National End of Life Care Programme (NEoLCP – parts of which now exist within NHS Improving Quality). One of the aims of the NEoLCP was to enable people to die in their preferred place, where possible. In order to achieve this, Advance Care Planning was encouraged and the use of tools such as Preferred Priorities for Care, a document held by individuals and stating their care preferences, potentially including the place they would like to die [7-9].

Evidence that such initiatives are impacting on place of death in the UK is starting to accumulate, with a recent study of deaths between 2004 and 2010 reporting a sustained trend towards an increasing proportion of home deaths, with corresponding decreases in hospital deaths in cancer patients [10]. This change has also occurred in haematological malignancies, although the decrease in hospital deaths is not as marked as for other cancers [11,12]. Importantly, the UK’s National Cancer Intelligence Network confirmed that the excess of hospital deaths for patients with haematological cancers persisted throughout 2001–2009 (68% for haematological cancers *versus* 47% for other cancers) [11].

There is a substantial literature examining place of death across various disease groups; but whilst there is speculation and anecdote regarding the reasons for the excess of hospital deaths in patients with haematological cancers, this has not previously been analysed by disease sub-type or time from diagnosis to death. Haematological malignancies account for one in ten of all cancers in the developed world however [13,14], and so their effective management is of particular importance not only to patients, but also health service managers and commissioners, who may be funding end-of-life care in inappropriate, acute hospital settings. Further information relating to the factors associated with hospital death will promote understanding and highlight ‘at-risk’ groups of patients who may benefit from early interventions to enable them to die in their preferred place, where possible. The aim of this study was to examine place of death in patients with haematological malignancies and variations related to diagnostic sub-type and time from diagnosis to death.

## Methods

This study was conducted within the infrastructure of the Haematological Malignancy Research Network (http://www.hmrn.org), an on-going patient cohort in the north of England, covering a population of 3.6 million that is broadly representative of the UK as a whole [15,16]. Established in 2004, HMRN is a collaboration between the clinical haematology network, researchers at the University of York and the Haematological Malignancy Diagnostic Service (http://www.hmds.info), which diagnoses all haematological malignancies in the study area, coding to the latest WHO classification scheme, ICD-0-3 [17]. More than 2,000 patients are registered annually and diagnostic, demographic, prognostic, treatment and outcome data are routinely abstracted from their medical records to clinical trial standards. All HMRN patients are ‘flagged’ for death via linkage to national data sources. HMRN has full ethical approval and Section 251 exemption to collect data for audit and research purposes, in addition to statutory governance approvals. The current study includes all adults (≥18 years) newly diagnosed with a haematological malignancy between 1st September 2004 and 31st August 2010, who died on or before 31st August 2012; thus giving a minimum follow-up time of two years and a maximum of eight years.

In the UK, hospitals provide generalist and/or specialist services to address a wide range of healthcare needs; hospices specifically focus on the provision of palliative and end-of-life care for people with life-limiting conditions; and nursing homes are residential institutions where healthcare is provided for people whose needs prevent them from living independently in their own homes. We examined the proportion of people dying in each of these settings, as well as those dying in their own home and examined differences by diagnostic sub-type. Time-to-death was also explored within specific time-intervals, ranging from less than one month to greater than one year, and variations were examined by place of death overall. Finally, time-to-death was explored by disease sub-type, specifically within the context of the hospital deaths. Data analyses was carried out in SAS version 9.3 [18] using standard descriptive methods. Odds ratios and corresponding 95% confidence intervals were estimated for hospital and non-hospital deaths using logistic regression; these were adjusted for age at diagnosis, sex and diagnosis. The likelihood Ratio Test for trend was used to examine patterns over the follow-up period.

## Results

10,325 patients aged 18 years and over were diagnosed with a haematological cancer between 1st September 2004 and 31st August 2010. The most common sub-types were diffuse large B-cell lymphoma (n = 1604), myeloma (n = 1399), chronic lymphocytic leukaemia (n = 1279) and myeloproliferative neoplasms (n = 1187) (Table 1). The median age at diagnosis was 70.9 years, reflecting the late age of disease onset of most of these cancers; the exceptions being Hodgkin lymphoma (median age 44 years) and acute lymphoblastic leukaemia (median age 47 years).

**Table 1:** Characteristics of patients and place of death

**Diagnosis**^**1**^	**Diagnosed (01/09/04-31/08/10)**		**Deceased (by 31/08/12)**	**Place of death**			
	**N**	**Median age at diagnosis**	**N (% of those diagnosed)**	**Hospital**	**Home**	**Nursing home**	**Hospice**
				**N (% of deaths)**	**N (% of deaths)**	**N (% of deaths)**	**N (% of deaths)**
**Total**	10325	70.9	4829 (46.8)	3183 (65.9)	753 (15.6)	532 (11.0)	361 (7.5)
Myeloma	1399	73.0	887 (63.4)	572 (64.5)	139 (15.7)	107 (12.0)	69 (7.8)
Diffuse large B-cell lymphoma	1604	70.4	831 (51.8)	535 (64.5)	122 (14.7)	94 (11.3)	80 (9.6)
Myelodysplastic syndromes	794	76.0	595 (74.9)	422 (70.9)	94 (15.8)	44 (7.4)	35 (5.9)
Acute myeloid leukaemia	722	71.1	573 (79.4)	413 (72.1)	81 (14.1)	41 (7.2)	38 (6.6)
Chronic lymphocytic leukaemia	1279	71.6	424 (33.2)	276 (65.1)	74 (17.5)	50 (11.8)	24 (5.6)
Myeloproliferative neoplasms	1187	71.2	283 (23.8)	161 (56.9)	50 (17.7)	53 (18.7)	19 (6.7)
Marginal zone lymphoma	625	72.2	229 (36.6)	141 (61.6)	28 (12.2)	42 (18.3)	18 (7.9)
Lymphoproliferative disorder NOS^2^	378	77.0	165 (43.7)	101 (61.2)	26 (15.8)	30 (18.2)	8 (4.8)
Follicular lymphoma	656	64.5	148 (22.6)	91 (61.5)	29 (19.6)	16 (10.8)	12 (8.1)
Hodgkin lymphoma	576	44.4	126 (21.9)	94 (74.6)	17 (13.5)	10 (7.9)	5 (4.0)
Mantle cell lymphoma	173	74.0	123 (71.1)	66 (53.7)	24 (19.5)	13 (10.6)	20 (16.2)
T-cell lymphoma	206	64.9	117 (56.8)	83 (70.9)	20 (17.1)	5 (4.3)	9 (7.7)
Chronic myelomonocytic leukaemia	127	77.4	89 (70.1)	64 (71.9)	16 (18.0)	7 (7.9)	2 (2.2)
Acute lymphoblastic leukaemia	112	47.5	77 (68.8)	53 (68.8)	10 (13.0)	4 (5.2)	10 (13.0)
Primary myelofibrosis	88	74.1	49 (55.7)	34 (69.4)	8 (16.3)	3 (6.1)	4 (8.2)
Chronic myeloid leukaemia	203	59.2	44 (21.7)	32 (72.7)	4 (9.1)	5 (11.4)	3 (6.8)
Burkitt lymphoma	53	57.6	30 (56.6)	21 (70.0)	5 (16.6)	2 (6.7)	2 (6.7)
T-cell leukaemia	80	74.7	30 (37.5)	19 (63.3)	4 (13.3)	5 (16.7)	2 (6.7)
Hairy cell leukaemia	63	65.5	9 (14.3)	5 (55.6)	2 (22.2)	1 (11.1)	1 (11.1)

^1^Ordered by absolute number of deaths; ^2^Not otherwise specified.

4829 (46.8%) of the 10,325 patients in the cohort died on, or before, the 31st August 2012. As can be seen from Table 1, which is ordered by the absolute number of deaths, the sub-types with the greatest number of deaths were myeloma (n = 887), diffuse large B-cell lymphoma (n = 831), myelodysplastic syndromes (n = 595) and acute myeloid leukaemia (n = 573). Across all diseases, the most common place of death was hospital (65.9%), followed by home (15.6%), nursing home (11%) and hospice (7.5%). Some variation was seen in place of death by diagnosis, with the highest proportion of hospital deaths overall occurring in patients with Hodgkin lymphoma, chronic myeloid leukaemia and acute myeloid leukaemia. The proportion of hospital deaths was generally somewhat lower in patients with myeloma and non-Hodgkin lymphoma. Nursing home deaths were more common in those with very indolent diseases such as myeloproliferative neoplasms, marginal zone lymphoma and lymphoproliferative disorders. Hospice deaths were relatively infrequent across all haematological disease sub-types, but somewhat more common in mantle cell lymphoma and acute lymphoblastic leukaemia. Home deaths occurred more often in patients with follicular and mantle cell lymphomas.

Place of death varied with time from diagnosis to death (Table 2), with the highest proportion of hospital deaths (87.7%) occurring within the first month of diagnosis. Accompanied by a gradual increase in home and hospice deaths, the proportion of hospital deaths fell to 71.6% between 1–3 months and then again to 61.7% between 3–6 months, remaining at around this level thereafter. Overall (Table 3), the odds of a non-hospital death was nearly three times greater if death occurred between 1–3 months after diagnosis compared to the first month of diagnosis (Odds Ratio (OR) 2.83, 95% Confidence Interval (95% CI) 2.12-3.78), increasing to almost five times greater one-year after diagnosis (OR 4.91, 95% CI 3.85-6.28). The likelihood of dying outside hospital increased significantly over the full eight year period of observation (OR 1.02, 95% CI 1.01-1.02, Likelihood Ratio Test for Trend P < 0.0001), and these associations remained after adjusting for age at diagnosis, sex and diagnosis.

**Table 2:** Variation in place of death and diagnostic sub-type by time from diagnosis to death

	**Time from diagnosis to death**					
	**Total**	**0-1 month**	**1-3 months**	**3-6 months**	**6 months-1 year**	**1 year +**
	**N (%)**	**N (%)**	**N (%)**	**N (%)**	**N (%)**	**N (%)**
**Total deaths**	4829 (100)	676 (100)	623 (100)	538 (100)	767 (100)	2225 (100)
**Place of death (total deaths)**						
Hospital	3183 (65.9)	593 (87.7)	446 (71.6)	332 (61.7)	494 (64.4)	1318 (59.2)
Home	753 (15.6)	42 (6.2)	76 (12.2)	90 (16.7)	133 (17.3)	412 (18.5)
Nursing home	532 (11.0)	23 (3.4)	61 (9.8)	72 (13.4)	74 (9.6)	302 (13.6)
Hospice	361 (7.5)	18 (2.7)	40 (6.4)	44 (8.2)	66 (8.6)	193 (8.7)
**Disease sub-type (hospital deaths)**^**1**^						
Myeloma	572 (18.0)	77 (13.0)	85 (19.1)	40 (12.1)	98 (19.8)	272 (20.6)
Diffuse large B-cell lymphoma	535 (16.8)	182 (30.7)	98 (22.0)	65 (19.6)	79 (16.0)	111 (8.4)
Myelodysplastic syndrome	422 (13.2)	27 (4.5)	55 (12.3)	68 (20.5)	85 (17.2)	187 (14.2)
Acute myeloid leukaemia	413 (13.0)	151 (25.5)	82 (18.4)	51 (15.3)	48 (9.7)	81 (6.1)
Chronic lymphocytic leukaemia	276 (8.7)	29 (4.9)	14 (3.1)	19 (5.7)	35 (7.1)	179 (13.6)
Myeloproliferative neoplasms	161 (5.1)	15 (2.5)	8 (1.8)	10 (3.0)	18 (3.6)	110 (8.3)
Hodgkin lymphoma	94 (2.9)	16 (2.7)	20 (4.5)	14 (4.2)	14 (2.8)	30 (2.3)
Follicular lymphoma	91 (2.8)	7 (1.2)	7 (1.6)	2 (0.6)	16 (3.2)	59 (4.5)
Acute lymphoblastic leukaemia	53 (1.7)	8 (1.3)	10 (2.2)	3 (0.9)	14 (2.8)	18 (1.4)
Others	566 (17.8)	81 (13.7)	67 (15.0)	60 (18.1)	87 (17.6)	271 (20.6)

^1^Ordered by absolute number of hospital deaths.

**Table 3:** Risk of hospital vs non-hospital death by time from diagnosis to death

		**Place of death**		**OR (95% CI)**^**1**^	**Adjusted OR (95% CI)**^**1**^
	**Total**	**Hospital**	**Non-hospital**		
Time from diagnosis to death:	4829 (100)	3183 (65.9)	1646 (34.1)		
0-1 month	676 (100)	593 (87.7)	83 (12.3)	1	1
1-3 months	623 (100)	446 (71.6)	177 (28.4)	2.83 (2.12-3.78)	3.00 (2.24-4.01)
3-6 months	538 (100)	332 (61.7)	206 (38.3)	4.43 (3.32-5.91)	5.01 (3.74-6.71)
6 months – 1 year	767 (100)	494 (64.4)	273 (35.6)	3.95 (3.00-5.19)	4.59 (3.47-6.08)
1 year +	2225 (100)	1318 (59.2)	907 (40.8)	4.91 (3.85-6.28)	5.87 (4.53-7.60)

^1^Odds Ratios (OR) and corresponding 95% Confidence Intervals (95% CI) adjusted for age, sex and diagnosis.

**Figure 1:**
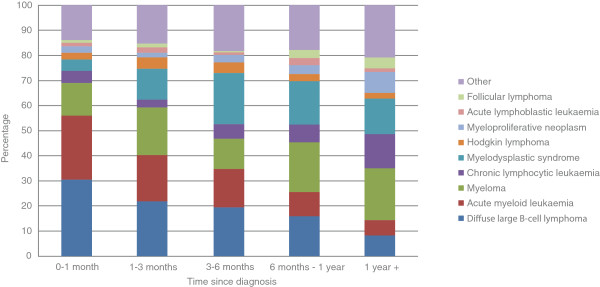
Time from diagnosis to death by diagnostic sub-type (hospital deaths only).

Among hospital deaths within the first month of diagnosis (early deaths), diffuse large B-cell lymphoma (30.7%), acute myeloid leukaemia (25.5%), and myeloma (13.0%) predominate (Table 2, Figure 1). The relative contribution of the different sub-types changed over time; with myeloma accounting for one in five hospital deaths occurring more than a year from diagnosis (20.6%), followed by myelodysplastic syndromes (14.2%) and chronic lymphocytic leukaemia (13.6%).

## Discussion

This study examined, for the first time, the relationship between place of death and time from diagnosis to death across distinct haematological malignancy sub-types. Over the eight years 2004–12, around two-thirds of all deaths within our cohort occurred in hospital, and this proportion was similar across most sub-types. Time from diagnosis to death was clearly a major determinant of place of death and, in this respect, sub-type was important. Almost 90% of patients dying within a month of diagnosis did so in hospital and these early deaths largely comprised patients with acute myeloid leukaemia, diffuse large B-cell lymphoma and myeloma. Patients surviving longer, and particularly beyond one year, were less likely to die in hospital. Our overall proportion of hospital deaths (66%) is consistent with the most recent UK national data (68%) [11]; and previous studies have also reported longer survival to be associated with deaths outside hospital [6,19], although none have examined these variables by specific haematological disease sub-type.

Emanating from within an established population-based patient cohort [15,16], the findings from this study are robust and it is likely that the patterns we observed are generalizable across the UK, and possibly to other countries with similar healthcare systems and cultural practices. Centralised diagnostics is a core aspect of our cohort, and all diagnoses are routinely coded by clinical staff to the latest WHO scheme, currently ICD-0-3 [17]. Using these data, we were able to examine place of death by specific diagnostic sub-types, rather than adopting the more common approaches, in which these diseases have been examined either as part of the total group of cancer patients, as a component of an ‘other’ category, or within combinations of all, or certain haematological malignancies [20-25].

Importantly, this report has highlighted specific groups of patients that are ‘at risk’ of hospital death. Haematological diseases are complex, with heterogeneous pathways from diagnosis to death. Patients with acute myeloid leukaemia, diffuse large B-cell lymphoma and myeloma that die soon after diagnosis are likely to die in hospital for a number of reasons relating to their specific disease pathways. Acute myeloid leukaemia, for example, is typically associated with aggressive presentation and rapid progression. Initial hospital administered chemotherapy treatment can cause severe toxicity, including neutropenic sepsis, and there is a real possibility that sudden deterioration and death could occur as a consequence of either disease progression or the side-effects of treatment. While diffuse large B-cell lymphoma and myeloma may not always present as acutely, and treatment is often administered on an out-patient basis, it can be associated with similar toxicities to those seen with acute myeloid leukaemia. Such episodes may also result in hospital admissions, which can have the propensity end with sudden, unexpected death.

Hospital deaths in this acute context, occurring relatively unexpectedly and still within the framework of a curative/life prolonging approach to care, are arguably less avoidable than hospital deaths that occur after a longer illness. In the latter situation, where the disease has reached an anticipated terminal stage, it might be expected that there has been sufficient time to raise and discuss the subject of preferred place of care and death, and to make the appropriate arrangements. However, our results show that large numbers of patients with indolent diseases and longer survival also die in hospital, indicating that additional factors also seem to contribute to place of death.

One explanation, for this is that hospital may be the preferred place of death for some, although this has never previously been explored in patients with haematological malignancies, despite differences in the trajectories and treatment pathways of these diseases compared to other illnesses. Patients may, however, be under the care of the haematology team at the hospital over many years [15], during which time they often have close and sustained contact with the clinical team managing their care, leading to strong mutual relationships. A further issue to consider is that whilst we were able to identify deaths that occurred in hospital as part of this study, it is possible that some patients may have died in these settings, but on palliative care wards or in General Practitioner (GP) led local hospital wards, where the approach to care is palliative and similar to that of a hospice.

Other reasons for hospital deaths in patients with longer survival include the remitting/relapsing course of many of these diseases, which may result in treatments being given (sometimes for symptomatic relief) in the later stages of the pathway. The associated toxicities from this can again result in sudden and unexpected death, without the time to discuss place of death or plan home-discharge if this is the preferred and feasible option. Importantly, there is often a supportive element to the treatment of these diseases, including the transfusion of blood products for bone marrow failure and the administration of intravenous antibiotics for infection, both of which are generally delivered in an in-patient setting, and from which (particularly during the terminal stage of illness) the patient may not recover, thus resulting in hospital death.

Furthermore, the complexities associated with the trajectory of these diseases mean that identifying the transition from a curative to palliative approach to care can be challenging. As a consequence, clinicians have been criticised for continuing to treat advanced disease that is unlikely to respond to treatment, rather than recognising that the transition should occur [26,27]. Further understanding of such transitions, as well as evidence to guide both patient and clinician treatment decisions (including quality of life, biological and economic data) would contribute to early treatment/care planning and may lead to a reduction in the number of hospital deaths.

Despite these difficulties increasing proportions of deaths are occurring outside hospital in patients that survive longer. As well as facilitating a defined transition and advance end-of-life care planning, longer survival is generally associated with an increased likelihood of receiving input from specialist palliative care services, which is reported to facilitate home deaths for people with both haematological cancers and solid tumours [6,28-32]. It is not always clear whether palliative care referrals are made to facilitate home death when this is the preferred place, or whether involvement of the palliative care team facilitates discussions about end-of-life care, and exploration of the feasibility of home-death. The impact of such referrals is, however, clear and important.

Whilst we did not examine this in the present study, one factor worth considering in future projects is the impact of time-to-diagnosis on both survival and place of death. It is recognised that the time interval between symptom onset and diagnosis in patients with haematological malignancies, and especially myeloma and lymphoma, is often particularly prolonged [33-35]. Compared to people with other cancers such patients are more likely to present to hospital for the first time as an emergency, which is itself associated with poorer survival [36]. Delayed diagnosis is also reported to lead to increased complications (such as anaemia, bone disease and renal failure in myeloma) at diagnosis [37] possibly requiring hospitalisation. In such circumstances, we would anticipate that some of the patients experiencing protracted time-to-diagnosis (especially those with myeloma and lymphoma) are included in the group that die soon after diagnosis and in hospital. Importantly, however, the situation is far from straight-forward and no single explanation is likely to fit all disease types; the early hospital deaths we identified will undoubtedly also include patients that experienced rapid symptom onset and diagnosis, particularly those with acute myeloid leukaemia.

Haematological malignancies are commoner in older people, and patients with these diseases may also have significant or multiple co-morbidities that contribute to their death. Although we did not examine cause of death within our study, the influence of comorbidities may be important and could in fact have a greater influence on place of death than the haematological malignancy itself; and this is particularly so for patients with more indolent disease sub-types. Whilst deaths from comorbidities undoubtedly occur in all the locations we examined, it is interesting to note the high proportion of nursing home deaths in patients with diseases that are usually monitored rather than actively treated, such as lymphoproliferative disorders (median age at diagnosis 77 years). It is likely that these patients are nursing home residents due to other comorbid/age-related factors, rather than solely because of their haematological malignancy.

Existing research has reported that home deaths are more likely to occur when home is stated as the patient’s preferred place of care [6]. Within this study, it was not our intention to explore preferred place of care and death. This is because, along with other researchers, we have previously found great variation in the frequency and methods of documentation of such conversations, [38]. Although standardised tools exist to facilitate discussion and documentation in primary care, we have not generally observed the use of these in secondary care settings; hand searching of hospital records for such information was beyond the scope of this present study.

## Conclusions

In the UK, a large proportion of patients with haematological malignancies die in hospital across all diagnostic sub-types. Time from diagnosis to death was clearly a major determinant of place of death and many patients that died within three months of diagnosis did so in hospital. This was closely related to disease sub-type, with early deaths occurring most notable in the more aggressive diseases. This is likely to be due to a combination of factors including acute presentation, rapid disease progression without transition to a palliative approach to care and complications of treatment. Nonetheless, hospital deaths also occurred frequently in indolent diseases, suggesting that other factors were likely to contribute to the large proportion of hospital deaths overall. This may include hospital being the preferred place of care, or indeed death being caused by comorbidities unrelated to the haematological malignancy, but requiring hospitalisation. More evidence is needed to fully understand these complex cancers.

## Study approvals

The Haematological Malignancy Research Network has ethical approval (REC 04/01205/69) from Leeds West Research Ethics Committee, R&D approval from each Trust in the Yorkshire and Humber and Yorkshire Coast Cancer Networks, and exemption from Section 251 (formally Section 60) of the Health & Social Care Act (2001) (PIAG 1-05(h)/2007).

## Competing interests

The authors declare that they have no competing interests.

## Authors’ contributions

The study was planned and instigated by DH, ER and AS; AS managed the data and directed data analysis; HW conducted data analysis; MH, and RP provided clinical input; DH, ER and AS wrote the first draft of the manuscript; all authors contributed to the final version of the manuscript. All authors read and approved the final manuscript.

## Pre-publication history

The pre-publication history for this paper can be accessed here:

http://www.biomedcentral.com/1472-684X/12/42/prepub

## References

[B1] Higginson IJ Sen-Gupta GJ Place of care in advanced cancer: a qualitative systematic literature review of patient preferences J Palliat Med 2000 3 287 300 15859670 10.1089/jpm.2000.3.287

[B2] Gomes B Calanzani N Higginson IJ Local preferences and place of death in regions within England 2010 2011 London: Cicely Saunders International

[B3] Gomes B Calanzani N Gysels M Hall S Higginson IJ Heterogeneity and changes in preferences for dying at home: a systematic review BMC Palliative Care 2013 12 7 23414145 10.1186/1472-684X-12-7 PMC3623898

[B4] Cohen J Bilsen J Addington-Hall J Löfmark R Miccinesi G Kaasa S Onwuteaka-Philipsen B Deliens L Population-based study of dying in hospital in six European countries Palliat Med 2008 22 702 710 18715968 10.1177/0269216308092285

[B5] Howell DA Roman E Cox H Smith AG Patmore R Garry AC Howard MR Destined to die in hospital? Systematic review and meta-analysis of place of death in haematological malignancy BMC Palliat Care 2010 9 9 20515452 10.1186/1472-684X-9-9 PMC2892433

[B6] Gomes B Higginson IJ Factors influencing death at home in terminally ill patients with cancer: systematic review BMJ 2006 332 515 521 16467346 10.1136/bmj.38740.614954.55 PMC1388126

[B7] Department of Health End of life care strategy: promoting high quality care for adults at the end of their life 2008 London: Department of Health

[B8] National End of Life Care Programme: Advance care planning: a guide for health and social care staff 2008 London: Department of Health

[B9] Department of Health End of life care programme: preferred priorities for care 2007

[B10] Gomes B Calanzani N Higginson IJ Reversal of the British trends in place of death: time series analysis 2004–2010 Palliat Med 2012 26 102 107 22258367 10.1177/0269216311432329

[B11] National Cancer Intelligence Network Where do patients with blood cancers die? NCIN data briefing 2011 London: NCIN

[B12] Gao W Ho YK Verne J Glickman M Higginson IJ on behalf of the GUIDE_Care project Changing patterns in place of cancer death in England: a population-based study PLoS Med 2013 127 917 2893

[B13] Ferlay J Shin H-R Bray F Forman D Mathers C Parkin DM Estimates of worldwide burden of cancer in 2008: GLOBOCAN 2008 Int J Cancer 2010 10.1002/ijc.25516 21351269

[B14] Jemal A Siegel R Ward E Hao Y Xu J Murray T Thun MJ Cancer statistics, 2008 CA Cancer J Clin 2008 58 71 96 18287387 10.3322/CA.2007.0010

[B15] Smith A Roman E Howell D Jones R Patmore R Jack A The Haematological Malignancy Research Network (HMRN): a new information strategy for population based epidemiology and health service research Br J Haematol 2010 148 739 753 19958356 10.1111/j.1365-2141.2009.08010.x PMC3066245

[B16] Smith A Howell D Patmore R Jack A Roman E Incidence of haematological malignancy by sub-type: a report from the Haematological Malignancy Research Network Br J Cancer 2011 105 1684 1692 22045184 10.1038/bjc.2011.450 PMC3242607

[B17] Swerdlow SH Campo E Harris NL Jaffe ES Pileri SA Stein H Thiele J Vardiman JW WHO classification of tumours of haematopoietic and lymphoid tissues 2008 4 Lyon, France: IARC Press

[B18] SAS Institute Inc SAS statistical analysis software 2011 Cary, NC, USA: SAS Institute Inc

[B19] Murray MA Fiset V Young S Kryworuchko J Where the dying live: a systematic review of determinants of place of end-of-life cancer care Oncol Nurs Forum 2009 36 69 77 19136340 10.1188/09.ONF.69-77

[B20] Gallo WT Baker MJ Bradley EH Factors associated with home *versus* institutional death among cancer patients in Connecticut J Am Geriatr Soc 2001 49 771 777 11454116 10.1046/j.1532-5415.2001.49154.x

[B21] Hunt R McCaul K A population-based study of the coverage of cancer patients by hospice services Palliat Med 1996 10 5 12 8821183 10.1177/026921639601000103

[B22] Hunt R Bonett A Roder D Trends in the terminal care of cancer patients: South Australia, 1981–1990 Aust N Z J Med 1993 23 245 251 7688953 10.1111/j.1445-5994.1993.tb01725.x

[B23] McCusker J Where cancer patients die: an epidemiologic study Public Health Rep 1983 98 170 176 6856741 PMC1424429

[B24] Burge F Lawson B Johnston G Trends in the place of death of cancer patients, 1992–1997 CMAJ 2003 168 265 270 12566330 PMC140467

[B25] Moinpour CM Polissar L Factors affecting place of death of hospice and non-hospice cancer patients Am J Public Health 1989 79 1549 1551 2817170 10.2105/ajph.79.11.1549 PMC1349812

[B26] McGrath P Are we making progress? Not in haematology! Omega: The Journal of Death and Dying 2002 45 331 348 10.2190/KU5Q-LL8M-FPPA-LT3W 15106653

[B27] McGrath P Palliative care for patients with hematological malignancies–if not, why not? J Palliat Care 1999 15 24 30 10540795

[B28] Grande GE Addington-Hall JM Todd CJ Place of death and access to home care services: are certain patient groups at a disadvantage? Soc Sci Med 1998 47 565 579 9690840 10.1016/s0277-9536(98)00115-4

[B29] Ansell P Howell D Garry A Kite S Munro J Roman E Howard M What determines referral of UK patients with haematological malignancies to palliative care services? An exploratory study using hospital records Palliat Med 2007 21 487 492 17846088 10.1177/0269216307082020

[B30] Houttekier D Cohen J Van den Block L Bossuyt N Deliens L Involvement of palliative care services strongly predicts place of death in Belgium J Palliat Med 2010 13 1461 1468 21133810 10.1089/jpm.2010.0279

[B31] Higginson IJ Wilkinson S Marie Curie nurses: enabling patients with cancer to die at home Br J Community Nurs 2002 7 240 244 12048497 10.12968/bjcn.2002.7.5.10359

[B32] Gomes B Calanzani N Curiale V McCrone P Higginson IJ Effectiveness and cost-effectiveness of home palliative care services for adults with advanced illness and their caregivers Cochrane Database Syst Rev 2013 6 CD007760 10.1002/14651858.CD007760.pub2 PMC4473359 23744578

[B33] Howell DA Time-to-diagnosis and symptoms of myeloma, lymphomas and leukaemias: a report from the Haematological Malignancy Research Network BMC Haematology 2013 13 9 10.1186/2052-1839-13-9 PMC4176985 24238148

[B34] Lyratzopoulos G Abel GA McPhail S Neal RD Rubin GP Measures of promptness of cancer diagnosis in primary care: secondary analysis of national audit data on patients with 18 common and rarer cancers Br J Cancer 2013 108 90 686 10.1038/bjc.2013.1 PMC3593564 23392082

[B35] Lyratzopoulos G Neal RD Barbiere JM Rubin GP Abel GA Variation in number of general practitioner consultations before hospital referral for cancer: findings from the 2010 National Cancer Patient Experience Survey in England Lancet Oncol 2012 13 353 365 22365494 10.1016/S1470-2045(12)70041-4

[B36] Elliss-Brookes L McPhail S Ives A Greenslade M Shelton J Hiom S Richards M Routes to diagnosis for cancer – determining the patient journey using multiple routine data sets Br J Cancer 2012 107 1220 1226 22996611 10.1038/bjc.2012.408 PMC3494426

[B37] Kariyawasan CC Hughes DA Jayatillake MM Mehta AB Multiple myeloma: causes and consequences of delay in diagnosis QJM 2007 100 635 640 17846059 10.1093/qjmed/hcm077

[B38] Cox K Moghaddam N Almack K Pollock K Seymour J Is it recorded in the notes? Documentation of end-of-life care and preferred place to die discussions in the final weeks of life BMC Palliative Care 2011 10 18 22053810 10.1186/1472-684X-10-18 PMC3227605

